# Lysophosphatidic acid selectively modulates excitatory transmission in hippocampal neurons

**DOI:** 10.1186/s13578-025-01458-y

**Published:** 2025-08-12

**Authors:** Nicola Brandt, Arne Battefeld, Olga Suckau, Konstantin Stadler, Bhumika Singh, Pei Zhang, Junken Aoki, Jerold Chun, Christian Henneberger, Rosemarie Grantyn, Johannes Vogt, Robert Nitsch, Ulf Strauss, Anja U. Bräuer

**Affiliations:** 1 Research Group Anatomy, School of Medicine and Health Sciences, Carl von Ossietzky University Oldenburg, 26129 Oldenburg, Germany; 2 Research Center for Neurosensory Science, Carl von Ossietzky University Oldenburg, Oldenburg, Germany; 3 Institute of Cell Biology and Neurobiology, Charité - Universitätsmedizin Berlin, 10117 Berlin, Germany; 4 Univ. Bordeaux, CNRS, IMN, UMR 5293, F-33000 Bordeaux, France; 5 SensLab GmbH, 12159 Berlin, Germany; 6 NTNU - Norwegian University of Science and Technology, Trondheim, Norway; 7 Institute of Physiology, Charité - Universitätsmedizin Berlin, 10117 Berlin, Germany; 8 Hsuanyeh Law Group, Boston, MA 02108 USA; 9 Graduate School of Pharmaceutical Science, University of Tokyo, 7-3-1, Hongo, Bunkyo-Ku, Tokyo, 113-0033 Japan; 10 The Scripps Research Institute, 10550 North Torrey Pines Read, La Jolla, San Diego, CA 92037 USA; 11 Institute of Cellular Neurosciences I, Medical Faculty, University of Bonn, 53127 Bonn, Germany; 12 Department of Molecular and Translational Neuroscience, Institute of Anatomy II, University of Cologne, Faculty of Medicine and University Hospital Cologne, 50931 Cologne, Germany; 13 Institute for Translational Neuroscience, Medical Faculty, Westfälische Wilhelms-University Münster, 48149 Münster, Germany

**Keywords:** LPA, Calcium imaging, LPA_2_ receptor, mEPCS, Hippocampus, Neuron

## Abstract

**Background:**

Lysophosphatidic acid (LPA) is a bioactive phospholipid that affects hippocampal excitatory synaptic transmission.

**Results:**

Here we provide in vitro evidence that LPA elicits intracellular calcium concentration ([Ca^2+^]_i_) transients by LPA_2_ receptor activation in primary cultured hippocampal mouse neurons. Downstream and via G_i_-coupling, this led to phospholipase C (PLC) activation, inositol (1,4,5) trisphosphate (IP_3_)-induced Ca^2+^ release (IICR) and voltage gated Ca^2+^ channel activation. In addition, we found that LPA elevated [Ca^2+^]_i_, not only in the soma but also in presynaptic terminals. This altered the frequency of spontaneous vesicle release specifically in excitatory synapses. However, against our expectations, LPA reduced the frequency of miniature excitatory postsynaptic currents. This was due to a depletion of releasable vesicles resulting from a slowed recycling. SynaptopHluorin based measurements indicated a transient augmentation of release followed by prolonged persistence of vesicles at the membrane. Concordant to our previous findings on ex vivo brain slices, LPA increased spontaneous glutamatergic vesicle release in Banker style astrocytic co-cultures. Our results indicate that pro-excitatory LPA effects critically depend on stable vesicle pools.

**Conclusions:**

Taken together, our data further support membrane derived phospholipids as active modulators of excitatory synaptic transmission.

**Supplementary Information:**

The online version contains supplementary material available at 10.1186/s13578-025-01458-y.

## Introduction

The central nervous system (CNS) is rich in membrane derived bioactive phospholipids such as LPA [1] and even cultured neurons produce nanomolar levels of LPA [2]. Serum levels of LPA range between 1 and 5 µM [3], but spatiotemporally mapping of LPA in specific CNS cell types remains challenging [1]. However, LPA production occurs upon activation of glutamate receptors [4] and CNS pathologies (trauma, severe haemorrhages) increase LPA tissue levels up to 10 µM [5]. Additionally, LPA and LPA mediator alterations appear to be a risk factor for several neuropsychiatric diseases and are implicated in the pathomechanism of e.g. Alzheimer`s disease or neuropathic pain [6].

As integral constituent of biological membranes, LPA is important in lipid metabolism, is involved in de novo synthesis of membrane phospholipids and changes biophysical membrane properties. LPA has also been linked to clathrin-dependent synaptic vesicle recycling [7, 8]. In addition, extracellular LPA is a potent bioactive signaling molecule partially via cell surface G-protein-coupled receptors (LPAR_1-6_) [1, 9, 10]. LPARs utilize intracellular signalling pathways, subsequently e.g. stimulating cell division, rearranging cytoskeleton, local transient Ca^2+^ changes and membrane movement [11]

As pioneering work uncovered, LPA affects synaptic signaling in a plethora of neurons (reviewed in [11]). In hippocampal ex vivo preparations, interfering with LPA levels influences glutamatergic transmission: perisynaptic modulation of LPA by autotaxin that is exclusively expressed in astrocytic processes at excitatory synapses and hydrolyzes lysophosphatidyl choline to LPA [12] or postsynaptic plasticity-related gene (PRG) 1 that controls phospholipids in the synaptic cleft [13] modulates excitatory transmission.

LPA_2_R’s presynaptic location at the excitatory synapse and the lack of LPA dependent excitability increase in LPA_2_R-deficient mice [13] suggest that LPA is involved in modulating glutamatergic transmission, however, evidence is mainly indirect so far. Most studies that link LPARs to function concern LPA_1_R, e.g. LPA_1_R activation in developing cortical neurons led to pertussis toxin (PTX) insensitive [Ca^2+^]_i_ increase from internal stores following TRCP channel activation [14]. However, LPA_1_R deficiency does not change synaptic function in the hippocampus [15] and even in motor-neurons where it reduces spontaneous synaptic release of glutamate, silencing LPA_1_R did not fully avoid LPA induced alterations [16]. LPAR expression changes under certain conditions e.g. after traumatic brain injury [17] that may include the preparation of acute brain slices and LPARs differ in various brain areas and stages of development, i.e. hippocampal neurons loose their LPA responsiveness during differentiation [14]. LPA_1_R and LPA_2_R were almost constitutively expressed during hippocampal development on the mRNA level [9, 18]. In line with expression studies [19], in brain cell cultures, LPA_1_R protein is mainly detected in oligodendrocyte lineage cells and in immature neurons. LPA_2_R, on the other hand, is predominantly expressed in mature hippocampal neurons and glial cells, which indicates the putative importance of LPA_2_R based modulation in neuronal networks [9, 18].

To address contradictory results of previous studies we asked whether LPA directly, i.e. without any interactions between LPA and extracellular matrix [20, 21], affects intracellular calcium concentration [Ca^2+^]_i_ and synaptic transmission in primary cultured hippocampal neurons that developed a functional network.

## Results

### LPA transiently increases [Ca^2+^]_i_ in pyramidal hippocampal neurons in culture predominantly via LPA_2_ receptors

**Fig. 1:**
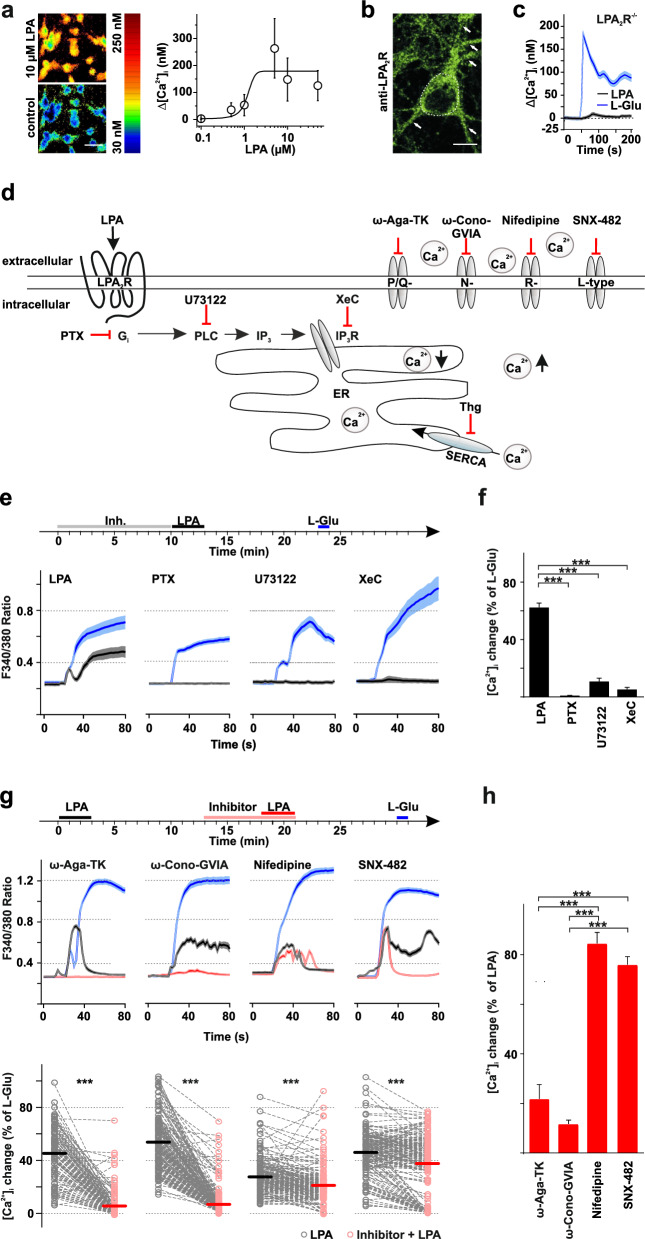
LPA induces increased intracellular Ca^2+^ levels via G_i_—signalling via LPA_2_-receptors and VGCC in primary cultured differentiated hippocampal mouse neurons. **a** *left:* Fura-2/AM imaging of primary cultured hippocampal mouse neurons (scale bar 20 µm). Application of 10 µM LPA increased [Ca^2+^]_i_ (*pseudo-coloured*). *right:* population data of average LPA-induced [Ca^2+^]_i_ differences (± SD) plotted against the respective LPA concentrations. The baseline [Ca^2+^]_i_ ranged from 57.6 to 172.2 nM and was on average 117.1 ± 23.3 nM (n = 125). Boltzmann fit revealed a dose-dependence. **b** Immunohistochemistry shows punctuated LPA_2_R expression (*as marked by arrows, for example; the dotted white line marks the soma*). Neurons were treated with 10 µM LPA for 30 min. Scale bar represents 10 µM. **c** The [Ca^2+^]_i_ increase triggered by 10 µM LPA was almost prevented in neurons from LPA_2_R^−/−^ mice, whereas glutamate (L-Glu, 100 µM) still increased [Ca^2+^]_i_. (**d**) Scheme of LPA_2_R-mediated intracellular signal transduction with respective inhibitors used in our experiments. (**e**) LPA induces [Ca^2+^]_i_ increase as measured with Fura-2/AM via G_i_-signalling cascade. *top panel:* schematic overview of the experimental setup and timeline. *lower panel:* representative traces of [Ca^2+^]_i_ responses of each condition in one experiment. Pre-incubation with 100 ng/ml Pertussistoxin (PTX) that blocks G_o_/G_i_, with 5 µM U-73122 that blocks phospholipase C (PLC) activation or with 1 µM XestosponginC (XeC) that blocks inositol (1,4,5) trisphosphate (IP3) receptors greatly reduced LPA-induced [Ca^2+^]_i_ increase in hippocampal neurons. Supramaximal glutamate was applied at the end of each experiment (e and g). Comparable glutamate application previously led to about 500 nM cytosolic [Ca^2+^]_I_ in hippocampal neurons [89]. (f) Quantification of averaged single neuron LPA peak values as percentage normalized to the respective glutamate peak values. Decrease of LPA induced [Ca^2+^]_i_ was observed with all inhibitors of the G_i_-signalling cascade. (g) Fura-2/AM imaging shows that Voltage gated Ca^2+^ channels (VGCCs) are also involved. *top panel:* schematic overview of the experimental setup and timeline. Representative traces of [Ca^2+^]_i_ responses of each condition of one experiment are shown in the middle panel. Inhibition of P/Q-type channels by 0.2 µM ω-Agatoxin-TK (ω-Aga-TK) and of N-type channels by 500 nM ω-Conotoxin-GVIA (ω-Cono-GVIA) reduced LPA-induced [Ca^2+^]_i_ increase. However, blocking L-type and R-type Ca^2+^ channels by 10 µM nifedipine or 1 µM SNX-842, respectively, did also interfere with LPA induced [Ca^2+^]_i_ increase but to a much lesser extent. The lower panel shows the quantification of the averaged LPA peak values normalized to glutamate peak values before (LPA) and after application of the respective inhibitor (inhibitor + LPA). Black (LPA) and red bars (inhibitor + LPA) indicate the mean. The occurrence of scatterd [Ca^2+^]_i_ peaks might be due to partially action potential driven network activity. (h) Quantification of the averaged inhibitor peak values normalized to LPA peak values. Decreases in LPA induced [Ca^2+^]_i_ were observed in neurons blocked with P/Q- and N-type channels inhibitors, and to a lesser extent with L-type and R-type channel blockers. For all n numbers, statistics and p values see Table S1

Treatment with LPA has been shown to increase [Ca^2+^]_i_ in a variety of cells [22]. However, the EC50 of LPA ranged from nanomolar to micromolar (7 nM —9.2 µM) depending on species and cell type [23–25], expression level of LPA receptors (e.g. overexpressed LPA_1-3_R, [26], LPA species depending on different acyl chain length and combination of expressed LPAR [27]. In the primary cultured hippocampal neurons that we investigated here, LPA increased [Ca^2+^]_i_ in a concentration-dependent manner with an EC50 of 1.2 µM (Fig. 1a), which is in the above mentioned range. The rise of [Ca^2+^]_i_ started within seconds, reached its peak within 75 – 85 s after addition of 10 µM LPA, gradually returned over the following minutes and could be re-activated to the same extent (Figure S1a), demonstrating that LPARs re-sensitize in our system.

To elucidate the role of LPA_2_R in mediating LPA induced transient [Ca^2+^]_i_ changes we first aimed to confirm its presence in primary hippocampal neurons in culture. LPA_2_R are expressed throughout cultured neurons (Fig. 1b) — as we previously showed for glutamatergic presynapses in brain slices [13] – here demonstrated by punctuated LPA_2_R signals in our immunohistochemical analysis. The LPA-induced [Ca^2+^]_i_ increase was mainly mediated by LPA_2_R because LPA-induced Ca^2+^ changes were clearly diminished in neurons from LPA_2_R^−/−^ mice (to ~ 5.9% of L-Glu induced [Ca^2+^]_i_ change; Fig. 1c). In contrast, glutamate application as a positive control still increased [Ca^2+^]_i_ levels (Fig. 1c) as in wildtype neurons. We, therefore, concentrated on LPA_2_R and subsequent signaling pathways.

### LPA induces somatic [Ca^2+^]_i_ increase via the G_i_-signaling cascade in hippocampal neurons

Both, influx from extracellular Ca^2+^ and release from internal stores, may cause the LPA-induced increase in [Ca^2+^]_i_. A specific LPAR and IP3 mediated Ca^2+^ release was first proposed in PC12 cells [28]. To elucidate the LPA_2_R-induced signaling in hippocampal neurons, we investigated the highlighted (Fig. 1d) steps of the LPA_2_R pathway. As many LPARs, LPA_2_R couple to different classes of G proteins, namely G_12/13_, G_q/11_, and G_i_, thereby mediating downstream signaling [29]. Ca^2+^ signaling can be modulated by G_i_ activation [30]. In line, blocking PTX-sensitive G proteins [31], when started 10 min before LPA exposure, prevented the LPA induced [Ca^2+^]_i_ increase (Fig. 1e, f). Since G_i_ (and G_q/11_) activates phospholipase C (PLC) we next antagonised PLC with 5 μM U-73122 for 5 min. Subsequent application of LPA only marginally increased [Ca^2+^]_i_ (Fig. 1e, f). PLC can either activate diacylglycerol (DAG)-dependent isoforms of protein kinase C (PKC) or increase [Ca^2+^]_i_ levels via inositol 1,4,5-triphosphate (IP_3_). Pre-treatment (5 min) with the IP_3_ receptor blocker Xestospongin C (XeC) inhibited the LPA-induced [Ca^2+^]_i_ increase (Fig. 1e, f). Because XeC might also inhibit voltage-dependent Ca^2+^ or K^+^ currents, we used thapsigargin in a different set of experiments. Thapsigargin is a cell-permeable inhibitor of the sarco/endoplasmic reticulum Ca^2+^-ATPase (SERCA) that induces release of intracellularly stored Ca^2+^ and precludes refilling of stores. In neurons that react to LPA, subsequent thapsigargin (5 μg/ml) application led to [Ca^2+^]_i_ elevations comparable to the increase induced by LPA. In addition, depletion of intracellular stores with thapsigargin prior to the LPA application nearly prevented the LPA induced [Ca^2+^]_i_ increase (Figure S1b). All neurons were viable as [Ca^2+^]_i_ increased in response to 100 mM L-glutamate after wash of LPA (Fig. 1e, g) or thapsigargin (Figure S1b). The lack of LPA-induced [Ca^2+^]_i_ increase when we beforehand inhibited either the IP_3_ receptor or SERCA suggests that [Ca^2+^]_i_ originates from the endoplasmic reticulum (IP_3_-sensitive Ca^2+^ stores). This means that G_i_-PLC-IP_3_ signaling is a major contributor to the Ca^2+^ increase in hippocampal neurons upon extracellular LPA increase.

Increases in [Ca^2+^]_i_ could also arise from influx through voltage-gated Ca^2+^ channels (VGCCs) that are critical for neurotransmission [32] and may open stochastically. We examined the role of VGCCs in LPA-induced Ca^2+^ increase in a series of experiments using various inhibitors (Fig. 1d). We long-term recorded neurons that responded to LPA (10 µM) application and investigated the relative effect of blockers on the second LPA application that followed 5 min pretreatment with either 0.2 µM ω-Agatoxin-TK (P/Q-type channels), 500 nM ω-Conotoxin-GVIA (N-type channels), 10 µM Nifedipine (L-type channels) or 0.1 µM SNX-482 (R-type channels). The LPA-induced [Ca^2+^]_i_ increase was reduced when neurons were pretreated with inhibitors of P/Q-type and N-type channels. This reduction was much less pronounced after L-type channel and R-type channel block (Fig. 1g, h).

Together, these results suggest that for LPA induced somatic [Ca^2+^]_i_ increase of hippocampal neurons internal stores are necessary, that VGCC may contribute to that increase and that in this case VGCCs complement one another.

### LPA selectively reduces excitatory transmission to hippocampal neurons

**Fig. 2:**
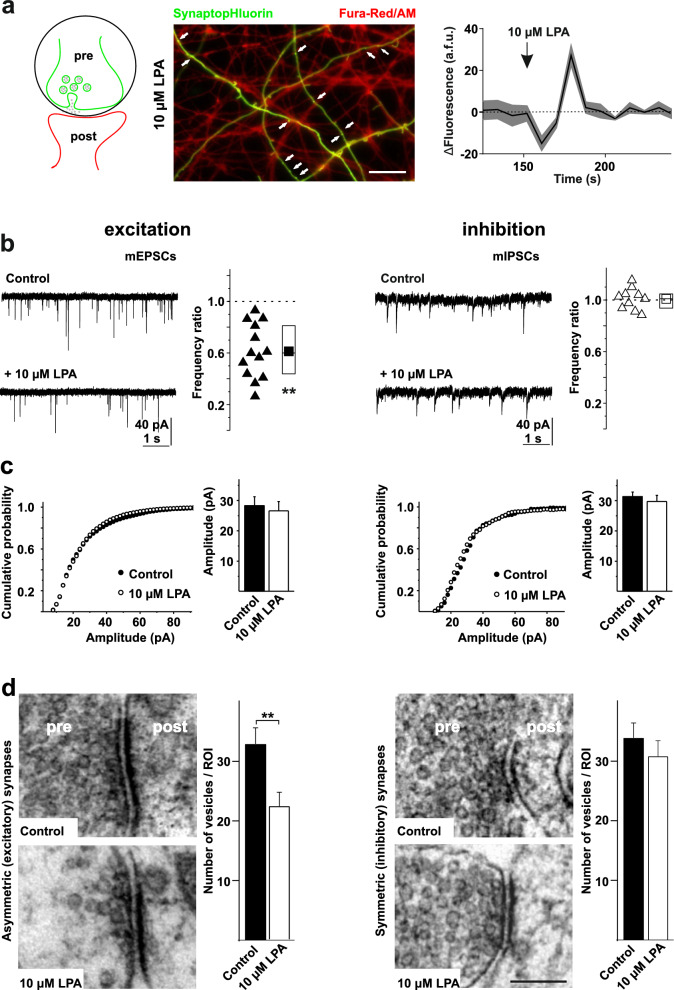
LPA acts on excitatory, but not inhibitory transmission in hippocampal neurons. **a** SynaptopHluorin responses and presynaptic Ca^2+^ increase elicited by LPA indicate LPA-induced exocytosis of synaptic vesicles in hippocampal neurons. *Left panel:* Schematic illustration of the pre- and postsynapse in hippocampal neurons expressing SynaptopHluorin (*green*) showing the ROI (*black circle*) representing the presynaptic terminal measured in this experiment. *Middle panel:* example of Fura-Red/AM loaded dissociated neurons (*red*) (axons and dendrites) expressing SynaptopHluorin (*green*) (transfected at 7–10 DIV and cultivated for further 3–5 DIV) at the peak of response elicited by 10 µM LPA. Arrows indicate the time of LPA application. LPA increased Ca^2+^ in close vicinity of exocytic sites in all likelyhood presynaptic terminals. After treatment, ROIs were picked around single synapses which showed exocytosis and fluorescence changes of Fura-Red/AM were calculated (*labelled with arrows*). *Right panel:* Time course of changes in fluorescence intensity of Fura-Red/AM at boutons expressing SytI-pH after stimulation with 10 µM LPA, showed LPA-induced Ca^2+^ increase in synaptic terminals. A representative tracing of fluorescence of four ROI is shown here. Note, that the limited acquisition frequency (0.2 Hz) – although sufficient for detecting the [Ca^2+^]_i_ increase — does not enable precise estimation of magnitude or kinetics. Time course and magnitude of [Ca^2+^]_i_ increase in the small presynaptic compartment might substantially differ from somatic [Ca^2+^]_i_ measurements (Fig. 1). Scale bar represents 5 µm. a.f.u. — arbitrary fluorescence unit. pre = presynapse; post = postsynapse. (**b**) Application of 10 µM LPA decreased the frequency of mEPSCs but not mIPSCs in primary hippocampal neurons. Representative mEPSCs or mIPSCs recorded before (*upper trace*) and after (*lower trace*) application of 10 µM LPA. Scale bars apply to all traces. Adjacent to the traces, the ratio of the frequencies of each neuron before and after LPA-treatment is plotted (*upward triangles*), showing the relative reduction, including median (*horizontal line*), mean (*square*) and 25-% and 75-% values. **c** Cumulative amplitude histogram constructed from > 2000 individual PSCs collected under control (*closed circles*) and LPA (*open circles*) conditions. On the right side the average miniature event amplitudes are plotted, respectively. **d** Electron microscopy analyses revealed a loss of vesicles in asymmetrical synapses after 10 µM LPA treatment (*left panel*), whereas symmetrical synapses showed no alteration in vesicle numbers *(right panel)*. Note, that we did not block action potentials in this trial. Scale bar represents 100 nm. pre = presynapse (axon terminal); post = postsynapse (dendrite). For all n numbers, statistics and p values see Table S1.

Ca^2+^ ions crucially regulate the rate of spontaneous neurotransmitter release [33]. Although IP_3_-sensitive stores are present in presynaptic terminals and participate in the generation of mEPSCs [34], a rise in somatic [Ca^2+^]_i_ is not necessarily accompanied by presynaptic [Ca^2+^]_i_ increase. Therefore we zoomed in on the vicinity of active presynaptic terminals as marked by SynaptopHluorin (Fig. 2a) to investigate a possible impact of LPA. A 5 – 15 s treatment with LPA led to [Ca^2+^]_i_ transients in the close surrounding of presynaptic terminals as indicated by Fura-Red/AM (Fig. 2a). To determine whether such LPA-induced, putatively presynaptic [Ca^2+^]_i_ increase influences spontaneous transmitter release in hippocampal neurons, we monitored miniature excitatory and inhibitory postsynaptic currents (mEPSCs and mIPSCs, respectively) in the presence of 2.5 µM tetrodotoxin (TTX). Comparing mEPSCs before and after LPA application revealed that LPA reduced their frequency (Fig. 2b) without changing the neuronal input resistance (Figure S1d). Amplitudes of mEPSCs did not differ between controls and LPA treated neurons (Fig. 2c). In addition, mEPSC amplitude distributions were not different with *vs.* without LPA, indicating that the effect on mEPSC frequency was not due to changes in detectability. In contrast to the postsynaptic LPA_1_R mediated reduction of GABAergic transmission on hypoglossal motoneurons [16] the frequency reduction in our experiments was specific for excitatory transmission, i.e. mIPSCs remained unchanged upon LPA application (Fig. 2b, c).

At sustained elevated [Ca^2+^]_i_ levels (above 5 µM in the Calyx of Held [35]) vesicle release rates are higher than those for recruitment – as a consequence vesicles are depleted. We next asked whether LPA treatment decreases the availability of vesicles at presynaptic sites by depletion. Ultrastructural analyses revealed a loss of vesicles in asymmetric (excitatory) synapses after treatment with 10 µM LPA for 10 min, whereas symmetric (inhibitory) synapses showed no alteration in vesicle number (Fig. 2d). Because we did not observe endosome like vesicles a dynamine/Adaptor Protein-2 (AP-2)/clathrin deficit [36] appears unlikely, in line with findings that kinetics of synaptic vesicle endocytosis are largely independent of clathrin and AP-2 and thus of classical clathrin mediated endocytosis in hippocampal neurons in culture [37]. We also did not observe dendrite retraction or cell rounding that could perturb synaptic transmission, in line with previous results [38, 39]. These data imply that although [Ca^2+^]_i_ transients occur in presynaptic terminals, spontaneous vesicle release is paradoxically decreased by LPA in excitatory synapses, while inhibitory synapses are not affected.

### Changes in mEPSC frequencies depend on LPA_2_R activation and (presynaptic) Ca^2+^ influx

**Fig. 3:**
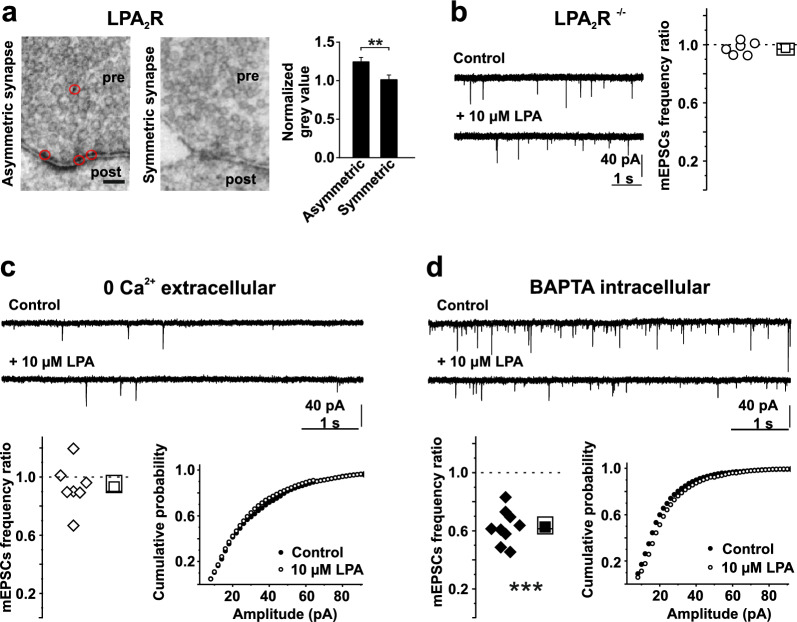
LPA effects depend on presynaptically expressed LPA_2_R and the presence of extracellular Ca^2+.^ (**a**) LPA_2_-receptor is located at asymmetrical (*left; red circles*), but not at symmetrical *(middle)* terminals as revealed by ultrastructural analyses (*right*). DAB reactions were estimated by intensity of grey value at the plasma membrane on the presynaptic side and normalized to controls (*right*). Scale bar represents 50 nm. pre = presynapse; post = postsynapse. Data shown in the histogram are means ± SEM. **b** Application of 10 µM LPA did not change the mEPSC frequency in hippocampal neurons without LPA_2_ (LPA_2_^−/−^), exemplified by traces (*left*) and given as population data (*right*). **c**, **d** mEPSC decrease after application of 10 µM LPA was prevented by (**c**) omitting Ca^2+^ in the external solution (i.e. pre- and postsynaptically) but not by (**d**) chelation of free Ca^2+^ in the postsynaptic neuron. *left*: typical mEPSCs in extracellular solution without Ca^2+^ (**c**) or with intracellularly applied fast Ca^2+^ chelator BAPTA (**d**), recorded before (*upper trace*) and 8 min after the application of LPA (*lower trace*). Scale bars apply to all traces. *Center:* population data on relative reduction of mEPSC frequency; *right:* cumulative amplitude histograms constructed from > 4000 individual PSCs collected under control (*closed circles*) and LPA (*open circles*) conditions. For all n numbers, statistics and p values see Table S1.

We previously identified expression of LPA_2_R specifically in presynaptic glutamatergic terminals in hippocampal sections [13]. Here we found somatic LPA_2_R expression in cultured hippocampal neurons (Fig. 1b). We next asked whether LPA_2_R are located in presynaptic axonal compartments in cultured hippocampal neurons, too. Ultrastructural analyses confirmed LPA_2_R presence in presynaptic terminals of asymmetrical (excitatory) synapses, whereas LPA_2_R were absent in symmetrical (inhibitory) synapses (Fig. 3a). Since the LPA-induced effect on [Ca^2+^]_i_ is presumably mediated by the LPA_2_R (Fig. 1c) we asked whether LPA_2_R is involved in spontaneous excitatory neurotransmission. We investigated the LPA effect on excitatory transmission in hippocampal neuronal cultures from LPA_2_R^−/−^ mice. There, LPA did not alter mEPSCs frequency or amplitude (Fig. 3b), suggesting that the LPA effect on glutamatergic terminals is primarily mediated by LPA_2_R.

Several lines of evidence point to a presynaptic mechanism. Firstly, the amplitude of mEPSCs, that is commonly interpreted as postsynaptic measure determined by AMPA channel density, did not change under the influence of LPA (Fig. 2c). This also suggests that initial steps in the vesicle-fusion process are not affected [40]. Secondly, eliminating extracellular Ca^2+^ (i.e. preventing pre- and postsynaptic Ca^2+^ influx) blocked the LPA effect (Fig. 3c). In line with findings that VGCCs are not necessary for spontaneous vesicle release [41] we recorded mEPSCs, although less frequently. The latter strengthens the view that stochastic VGCC (R-type) openings are a major trigger [42], and together with our somatic finding this might (by analogy) point to modulation by spontaneous release of Ca^2+^ from ER stores [43]. In contrast, blocking [Ca^2+^]_i_ increase in the postsynaptic neuron by intracellular application of BAPTA did not interfere with the LPA-induced effect (Fig. 3d), indicating that the LPA does not act postsynaptically or retrogradely. Therefore, Ca^2+^ influx either triggers a downstream effector (since we cannot exclude store depletion after “prolonged” exposure to 0 Ca^2+^) or (if mEPSC are VGCC driven) directly affects the spontaneous vesicle release.

In summary, limiting presynaptic Ca^2+^ entry abolishes the LPA effect. This points to opening of either VGCC at or near resting membrane potential that have been shown to have a strong impact on resting [Ca^2+^]_i_ levels such as L-type [44], N- and P/Q type [45], R-type channels [42] or ORAI [46] as prerequisites of LPA action. The latter two have been shown to regulate spontaneous vesicle release.

### LPA prolongs vesicle reuptake

Contrary to our expectations, the increase in pre-synaptic [Ca^2+^] concentration reduced, instead of increased, spontaneous vesicle release. We hypothesize that LPA additionally disturbs vesicle recycling in cultured neurons resulting in a mismatch of exo/endocytosis of spontaneously released vesicles. The impact of such a mismatch is long known for evoked release [47] where increase in release probability was obscured by a concomitant decrease in pool size.

**Fig. 4:**
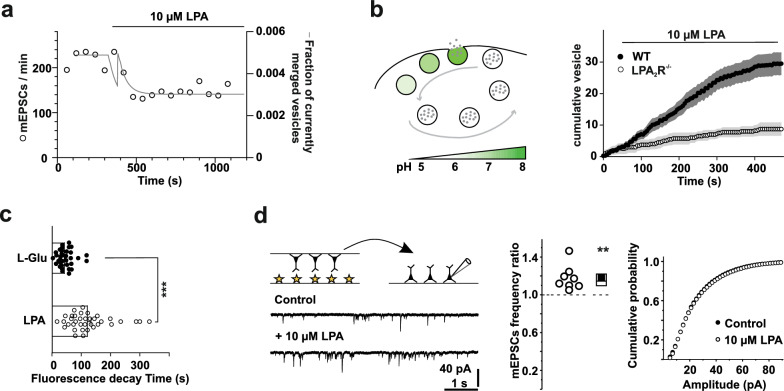
LPA modulates vesicle reuptake in primary hippocampal neurons and increases mEPSC frequencies in neuron—astrocyte co-cultures. **a** Single-pool mathematical three-state model of synaptic vesicle release (*solid line*) overlayed with mEPSC occurrence in hippocampal neurons *(open circles)* over 18 min. The model predicts that vesicle recycling rate is impacted faster than the vesicle release, therefore masking an LPA mediated increased vesicle release/increase of mEPSC frequency as previously shown in acute brain slices. For modelling details see methods. **b** *Left:* Scheme of vesicle cycling in presynaptic terminals using the SynaptopHluorin model. At rest, the fluorescence of SynaptopHluorin expressing vesicles is quenched due to the acidic pH in vesicle lumen. Upon fusion with the plasma membrane (exocytosis) the pH shifts towards basic because of the exposure of the lumen to the extracellular pH (~ 7.5). Subsequently, the fluorescence increases and is quenched again after endocytosis, when the pH returns to acidic levels. *Right:* LPA-induced accumulation of fused vesicles (SynaptopHluorin positive spots) is prominent at synaptic boutons of hippocampal neurons (WT, *solid circles*) but greatly reduced when no LPA_2_R is present (LPA_2_R^−/−^, *open circles*). **c** This accumulation was due to a prolonged membrane residual time of fused vesicles in the presence of LPA as estimated by the decay of SynaptopHluorin fluorescence, fit by a single exponential inset and compared to glutamate (L-Glu) stimulated vesicles. **d** *Upper panel:* Schematic representation of Banker’s style co-cultures. Hippocampal neurons (*upper coverslip*) were co-cultivated with astroglia (asterisks; *lower coverslip*) and the coverslips were flipped for measurements of hippocampal neurons. *Lower panel:* Representative current traces from hippocampal neurons held at -60 mV after Banker’s style co-cultivation. *Middle panel:* Population data on frequency ratio (frequency in LPA / frequency in buffer) of mEPSCs. LPA increased the frequency of mEPSCs. *Right panel:* Cumulative amplitude distribution and average amplitude remained comparable after application of 10 µM LPA. For all n numbers, statistics and p values see Table S1

LPA might either increase release and decrease endocytosois at the same time (suggestive for the same pathway, i.e. receptor/G-protein mediated) or staggered (suggestive for diverse mechanisms). To explore putative timelines we used an in silico system and adopted a single pool, three state model of a single presynapse [48]. This allows to vary the rate of exocytosis α (activation/release of vesicles), endocytosis σ and the recycling rate β (refilling of vesicles and transport to the pool of vesicles ready for release). We modelled the expected increase in mEPSC frequency with the initial parameters which resulted in a transient increase of merged membrane vesicles (Fig. 4a). However, if the near collapse of the recycling rate happens shortly before the increase of the exocytosis (here shown with a one minute delay) it would mask the transient increase in vesicle release. Such a modelled dynamic matches the observed mEPSC frequency decrease without initial increase.

Coordination of recycling, filling, and fusing of vesicles in terms of speed, fidelity, and sustainability needs precise orchestration of proteins and lipids, especially phospholipids [49] on multiple time scales [37]. Although membrane inserted LPA is involved in vesicle formation, too much LPA competitively prevents the effect, as previously shown in PC12 cells [8]. To visualize the influence of LPA on vesicle release and synaptic vesicle cycling we expressed a pHlourin that is linked to synaptobrevin (SynaptopHluorin) in cultured hippocampal neurons and performed life cell imaging. LPA stimulation led to a prominent accumulation of fused vesicles in hippocampal neurons at synaptic boutons, which was much smaller in neurons derived from LPA_2_ R^−/−^ knock-out mice (Fig. 4b), or, in other words, when LPA was unlikely to induce a [Ca^2+^]_I_ increase (Fig. 1c). We quantified the membrane residual time of fluorescent vesicle release sites and compared it to control stimulation with L-glutamate. The LPA-induced accumulation was associated with a prolonged membrane residual time of fused vesicles as estimated by the decay of SynaptopHluorin fluorescence compared to L-glutamate stimulated vesicles (Fig. 4c).

Together, our results suggest an imbalance in the rate of exo- and endocytosis of synaptic vesicles i.e. that vesicle recycling in the presence of LPA was interrupted or greatly decelerated.

### Astrocytes partially reverse LPA effects on mEPSCs

Astrocytes play key roles in formation, maturation, stabilization, and elimination of synapses [50] and they may do so (as in the case of thrombospondins [51]) after exposure of neurons to astrocytes or astrocyte conditioned medium. In particular, astrocytes maintain releasable vesicles in neurons [52]. Supporting this, basal mEPSC frequencies recorded in our Banker style astrocyte-neuron co-culture originated from hippocampal neurons of newborn mice were slightly elevated when compared to neurons cultured nominally free of astrocytes. By the use of Banker style cultures (i.e. by removal of the astrocytic layer before applying LPA) we excluded any secondary effect via astrocytes [21]. Under this condition and in line with our previous findings in acute hippocampal slices [13], LPA (10 μM) increased the mean frequency of mEPSCs without changing its amplitudes (Fig. 4d). These results imply that mechanisms are strictly neuronal, in principle comparable between culture and slice and that the transient presence of astrocytes may promote an LPA induced increase in synaptic transmission by stabilizing presynaptic vesicle pools or preventing LPA from slowing down vesicle recycling.

## Discussion

In this study we show that extracellular LPA elevation increases [Ca^2+^]_i_ mainly via LPA_2_R and LPA_2_R activation influences basal glutamate release in hippocampal pyramidal neurons that established a functional network. In our experiments, LPA induced a receptor-dependent intracellular signaling cascade most likely leading to a functional reduction of vesicle release as estimated by mEPSCs frequency. In pure neuronal cultures LPA controls glutamatergic vesicle availability by slowing of vesicle recycling. However, after astrocytic preconditioning of the neuronal cultures (Banker style cultures), presynaptic vesicle release was restored and increased after LPA application. As we observed LPA mediated [Ca^2+^]_i_ elevation, this result was initially expected, suggesting that astrocyte mediated maturation of primary neuronal cultures putatively reverse the imbalance in the rate of exo- and endocytosis.

Our results translate the pioneering findings of LPAR mediated dopamine release via IP3 induced Ca^2+^ rise [28] from adrenal neuronal crest cells to hippocampal principal neurons. The presented results suggest that varying conditions such as temperature and cellular environment dictate LPA effects and that LPAR differences might impede direct understanding when e.g. compared to LPA_1_R mediated signaling in hypoglossal motoneurons [16]. Redundant mechanisms in different cellular systems, for instance the LPA_1_R mediated signaling or the pathway alternative to NHERF2 and PLCβ activation as shown in different non-neuronal cell lines [53], as well as putative contribution of a variety of Ca^2+^ channels may point to the importace of extracellular LPA level changes for the synaptic transmission in general.

Several Ca^2+^-dependent pathways may regulate spontaneous neurotransmission [54], and may differ according to the synapse type. For instance, 50% of the mEPSCs are driven by calcium-induced calcium release in hippocampal cultures [43], whereas calcium-induced calcium release in parallel fibers is insignificant [55]. Corroborating our finding on undisturbed inhibitory spontaneous neurotransmission upon LPA application, store-operated Ca^2+^ entry, Ca^2+^ release from the ER [33] or changes in Orai protein levels [46] have been exclusively linked to excitatory neurotransmission. However, IP3 and ryanodine mediated transient [Ca^2+^]_i_ increase may not be major contributors to spontaneous glutamate release [34] and occur in spatially distinct domains [56]. Therewith, our finding on the contribution of VGCCs to LPA induced Ca^2+^ increase might gain relevance. VGCCs have been confirmed to underly spontaneous release of glutamatergic vesicles [56]. Nevertheless, we cannot exclude LPA induced IP3- or store operated Ca^2+^ entry after reducing ER Ca^2+^ levels. Unspecific membrane insertion of lipids, that induce exocytosis and reduce vesicle content in neuronal culture [57] or alterations in membrane tension [58], for instance by cholesterol insertion that in turn suppresses spontaneous vesicle cycling [59] are unlikely to account for the LPA mediated effects described here, because we observed receptor dependent LPA effects that were restricted to excitatory synapses.

An elevated mEPSC frequency is the expected consequence of a Ca^2+^ induced increase in the probability of spontaneous vesicle release. However, synaptic vesicle release depends on swift vesicle retrieval to prevent synaptic vesicle pool depletion [60] and involves rapid reuse of vesicles in hippocampal neurons [61]. Our data on vesicle loss or a prolonged membrane presence of vesicles support this role of endocytosis for fidelity of synaptic transmission [62] and are in line with a gradual decrease in vesicle release after blocking endocytosis [63].

Ca^2+^ may modulate vesicle recycling. Although it is not essential for vesicle retrieval [64], an increase in [Ca^2+^]_i_ above the inhibitory threshold of about 1 µM [Ca^2+^]_i_, shown to abolish endocytosis in synaptic terminals after evoked release [65], would be the most parsimonious explanation. For this, we need to assume that the mode of neurotransmission is determined by SNARE identity and their different Ca^2+^ sensitivity as well as that subsequent endocytosis modes are determined by [Ca^2+^]_i._ In that case, a presynaptic [Ca^2+^]_i_ increase might hamper proper endocytosis (or even switch endocytosis modes, i.e. its clathrin dependence). Increased presynaptic [Ca^2+^]_i_ may decelerate endocytosis, i.e. increase the proportion of vesicles retrieved by slow kinetics ([66], as bolstered by similar work [67]). Interestingly, presynaptic Ca^2+^ in the dialysed Calyx of Held could not been raised above 180 nM because of the substantial vesicle loss [68] (due to improper vesicle replacement?) insinuating a potentially lower threshold or a strong deceleration of endocytosis. Although generally assumed that it is advantageous for reuptake to use slowly changing global [Ca^2+^]_i_ signals for the purpose of tracing the history of previous electrical activity [68], a disturbance in the coupling of exo- and endocytosis either due to improper Ca^2+^ microdomains or to LPA interactions with proteins that link both processes [69] would also be possible. So far, a direct role for [Ca^2+^]_i_ in controlling presynaptic membrane homeostasis has been regarded unlikely [49], in particular for vesicles that fuse spontaneously [70]. Whether Ca^2+^ ions inhibit or promote endocytosis depends on particular conditions. In neuronal cultures, synapses with a higher baseline probability of vesicle fusion (that might implicate a higher [Ca^2+^]_i_) are slower to retrieve vesicles [71].

Alternatively, LPA_2_R signalling might interact with endocytic proteins in a way that decouples endo- from exocytosis when excitatory neurons increase their spontaneous release probability. Putative candidates would be proteins that are involved in the exocytosed Syt1-PiP_2_ mechanism [72]. More generally, LPA_2_R signalling might also interfere with regulated actin dynamics that is crucial for vesicle retrieval and reuse [37, 73, 74]. Actin perturbations were demonstrated to interfere with clathrin-independent endocytosis [75]. Other possibilities are synaptic vesicle proteins that recruit endocytic factors or modulate phospholipid content, e.g. synaptobrevin [33]. Notably, recycling (endocytic) pathways for spontaneous vesicle retrieval differ from the one for evoked release, i.e. operate clathrin-independent and unlikely involve canonical dynamin [33, 59, 70]. Therefore, it is unlikely that in our conditions of spontaneous vesicle release, LPA activated PLC-myosin light chain kinase as shown to underly presynaptic LPA_1_R mediated effects in evoked glutamatergic transmission in brainstem motoneurons [16]. However, we cannot fully exclude that a similar mechanism as stimulation of the actomyosin contractile apparatus contributes to the reduction of the readily releasable vesicle pool (Fig. 2d). Another possible explanation may relate to the reported temperature dependence of synaptic vesicle recycling, with higher recycling rates at physiological temperatures ([76], also reviewed by [77]). Performing electrophysiological analyses at 25 °C, we may have uncovered a role of LPA and of a soluble astrocytic factor in presynaptic terminals, which at physiological temperatures was compensated by higher vesicle rates. Furthermore, LPA membrane insertion may favor the membrane spread of vesicle components [78], thereby preventing synaptic vesicle protein concentration at endocytic sites [79], sorting into clathrin-independent carriers and location of fused membrane patches by the fast endocytic machinery [80].

When we cultured (i.e. preconditioned) neurons together with astrocytes, LPA could not override the known resistance to vesicle depletion in hippocampal neurons [81]. This result indicates that astrocytes prevent the LPA induced disturbance of proper exo-endocytotic coupling that our results in pure neuronal cultures suggested. Besides the hint that the responsible astrocytic factor is soluble, our experiments do not allow a further specification. However, soluble astrocyte-derived factors in development and maintenance of neuronal networks are well known [82] and several candidate molecules were identified as necessary for aspects of synapses maturation and refinement: 1. cholesterol in complex with ApoE stabilizes presynaptic function by controlling synaptic vesicle number and release probability. 2. Glypican 4 and 6 as well as TNFα positively regulate AMPA receptor localization. 3. Thrombospondins and SPARKCl1 influence synaptogenesis and regulate synaptic strength / induce or stabilize contacts between pre and postsynaptic terminals ([50], for review). Regardless their specific contribution, soluble astrocytic factors might support the maturation velocity of synapses and thus lead to more stable vesicle release. In our case, at DIV14, maturation without astrocytes might be incomplete, but with astrocytes synapses are matured and inherently more stable.

Our study puts LPA in line with other neuromodulators, such as nicotine, that take over signaling pathways and mediate activity-dependent plasticity in pyramidal neurons [83]. Because levels of spontaneous neurotransmission modulate network excitability, signalling, and different forms of plasticity [54], changes of spontaneous neurotransmission critically impact network firing properties and information processing. Consequently, aberrant spontaneous synaptic transmission is sufficient to cause developmental and epileptic encephalopathies in humans [84]. Manipulation of spontaneous neurotransmitter release by synaptic phospholipids could influence cortical hyperexcitability and E/I balance [12] and may be used as specific therapeutic strategy against neuropsychiatric disorders [85]. Our results are also important for further studying the role of LPA_2_R in presynaptic plasticity and maintenance of the fidelity of excitatory neurotransmission.

## Limitations

The mechanisms of LPA induced [Ca^2+^]_i_ increase we found in hippocampal neurons are somatic, involve multiple players and are not directly conveyable to the presynapse, i.e. compartment specific functions that might be mediated by different LPARs / pathways remain unresolved. In addition, the Ca^2+^ sensors we used are known to distort endogenous Ca^2+^ signal due to inherent buffering and kinetics [86]. Regarding the pathway, we did not exclude alternative explanations such as Gi/o coupled receptors that may lower presynaptic cAMP, thereby preventing the Ca^2+^ dependend recovery from depression [87]. For the proposed mismatch between vesicle release and reuptake, besides temperature effects, it might theoretically be that endocytosis is inherently marginally impaired due to culture conditions and the system collapses when release is increased although that has not been seen so far in this and many other culture systems. Finally, we did not identify the soluble astrocytic factor and did not investigate the influence of other CNS cells such as microglia or oligodendrocytes.

## Materials and methods

All resources are listed in the Supplementary Table 2.

### Animals

Neurons from male and female C57/Bl6 or BalbC mice were used for the experiments. Mice without LPA_2_R (LPA_2_R^–/–^) were genotyped as previously described [88]. Pregnant C57BL/6 or BalbC mice, obtained from our central animal facility, were kept under standard laboratory conditions (12 h (h) light/dark cycle; 55 ± 15% humidity; 24 ± 2 °C room temperature (RT) and water *ad libidum*) in accordance with German and European guidelines. Approval of experiments was obtained from 2010/63/EU; “Niedersächsisches Landesamt für Verbraucherschutz und Lebensmittelsicherheit” (33.19–42,502-04–18/2766) and the local ethical committee of Berlin (LAGeSO: T0108/11).

### Cell culture and lysophosphatidic acid

Primary hippocampal neurons were prepared from embryonic day 18 (± 0.5 days, E18). Hippocampi from several embryos were collected and washed twice in ice-cold HBSS (Hank's Buffered Salt Solution). The tissue was incubated in 4 ml HBSS and 400 µl trypsin for 15 min at 37 °C, resuspended in MEM (Modified Eagles Medium) plating medium supplemented with 10% horse serum, 0.6% glucose, 100 U/ml penicillin and 100 µg/ml streptomycin. Neurons were cultured on poly-L-lysine-coated glass coverslips in neurobasal A medium supplemented with 2% B27, and 0.5 mM glutamine, at a density of 120.000 to 150.000 × 10^5^ cells/well. For electrophysiological investigations penicillin and streptomycin were omitted. For calcium imaging experiments neurons were cultured at a density of 350.000 or 500.000 cells/well (6-well plates).

For neuron-astrocyte co-cultures [82] astroglial cells were prepared from hemispheres of P0—P2 mouse brains by enzymatic (2.5% Trypsin and 10 mg/ml DNAse I for 5 min at 37 °C) and mechanic disintegration in HBSS, strained (70 µm) in MEM, with 0,6% Glucose and 10% horse serum), centrifuged (300 × g for 5–10 min), resuspended in MEM and expanded. Medium was changed every 2–3 days. After 2 weeks astrocytes were plated in 12-well plates at 50.000 cells per well and preconditioned in Neurobasal A medium as above. Primary hippocampal neurons were prepared as described above, plated at 80.000 cells/well, incubated for 3 h and transferred upside down to the wells with the glial feeder layer.

Monounsaturated 1-Oleoyl-lysophosphatidic acid (18:1 LPA) that activates LPA receptors 1–6 [10] was dissolved in buffer containing (in mM): 50 HEPES, 138 NaCl, 2.7 KCl, 1 CaCl_2_, 1 MgCl_2_ and 1% fatty acid free bovine serum albumin (BSA), stocks were stored at –20 °C until use.

For calcium imaging experiments LPA was dissolved either in Krebs–Ringer’s solution, pH 7.4 (containing (in mM): 119 NaCl, 2.5 KCl, 1.0 NaH_2_PO_4_, 2.5 CaCl_2_ 2H_2_O, 1.3 MgCl_2_ 6H_2_O, 20 HEPES, 11 D-glucose) (G_i_-signaling cascade) or in Krebs–Ringer’s solution, where CaCl_2_ and MgCl_2_ were omitted (VGCC signaling cascade); stocks were stored at −20 °C until use.

### Electron microscopy

Immunohistochemistry was performed by using anti-LPA_2_. After treatment of the sections with 0.3% H_2_O_2_ in phosphate buffer (PB) for 30 min, the sections were blocked in a solution of 5% fetal calf serum (FCS)/PB at room temperature (RT). Cells were incubated with LPA_2_ antibody 1:100 in blocking solution overnight. An anti-rat biotin-conjugated secondary antibody was applied for 4 h at RT and subsequently visualized by performing a 3, 3’-diaminobenzidine (DAB) reaction with H_2_O_2_/PB. 1% cobalt chloride and 1% nickel ammonium sulphate were added to intensify the staining reaction. This step was omitted for cells assigned for visualization of symmetric and asymmetric synaptic terminals. Cells for electron microscopic analysis were postfixed in osmium tetroxide (1% in 0.1 M PB for 30 min), dehydrated and flat-embedded in Epon. Selected regions were re-embedded in plastic capsules and sectioned using a Reichert Ultracut, then mounted on single-slot grids coated with formvar film, stained with lead citrate, and viewed through a Zeiss electron microscope. All analyses were performed on single ultrathin sections of randomly selected synapses. The observer was blinded for treated and non-treated cells. Only synapses with intact synaptic plasma membranes with a recognizable pre- and postsynaptic density and clear synaptic vesicle membranes were analysed. Vesicles were counted in a region of interest (ROI) (500 nm x 250 nm), which was positioned at the presynaptic membrane. Data were collected from three independent experiments.

### Immunocytochemistry

Cultured primary neurons (DIV 14) were fixed in ice-cold 4% paraformaldehyde in 1 × phosphate-buffered saline (PBS) containing 15% sucrose for 20 min at RT. Following washing with 1 × PBS three times for 10 min neurons were subsequently permeabilized with 0.1% Triton X-100 and 0.1% sodium citrate in 1 × PBS for three minutes at 4 °C. After washing three times for 10 min in 1 × PBS cells were were incubated with 10% FCS in 1 × PBS for one hour at RT. Neurons were stained with monoclonal anti-LPA_2_ (1:250) (generated by J. Aoki) and incubated overnight at 4 °C. After washing three times for 10 min in 1 × PBS cells were incubated with secondary antibody goat anti-rat 488 Alexa Fluor conjugated (1:1000) at RT for one hour. All antibodies were diluted in 5% FCS in 1 × PBS. Coverslips were mounted on slides with Immu-Mount and used for microscopy.

### Ca^2+^ measurements

Neurons (DIV 7–11) were loaded with 2 µM Fura-2/AM in 1 × HEPES buffer (in mM: 137 NaCl, 5 KCl, 5.6 glucose, 20 HEPES, 0.59 KH_2_PO4, 0.56 Na_2_HPO4, 1.4 CaCl_2_, 0.9 MgSO_4_, 10 NaHCO_3_; pH 7.4) and incubated for 30 min at 37 °C. Neurons on coverslips were secured in a perfusion chamber mounted onto an inverted microscope equipped with a calcium imaging system (for detailed information see Supplementary Table 2). Cells were constantly superfused with Krebs–Ringer’s solution, containing (in mM): 119 NaCl, 2.5 KCl, 1.0 NaH_2_PO_4_, 2.5 CaCl_2_ 2H_2_O, 1.3 MgCl_2_ 6H_2_O, 20 HEPES, 11 D-glucose, pH 7.4) with 1 ml/min rate at 23–27 °C and a minimum of 3–5 min (for some experiments the neurons were superfused with HEPES-buffered HBSS (15 mM, pH7.4); Fig. 1a, c). Constant monitoring of free [Ca^2+^]_i_ concentration was performed using ratiometric measurements of fluorescence intensity at 340/380 nm. The cells were alternately excited at 340 nm and 380 nm for 30 ms at 2 s (sec) intervals (for some experiments 5 s intervals) and the emitted fluorescence at 510 nm was recorded.

After monitoring the baseline, LPA (10 µM; 0.1–50 µM) or L-glutamate (100 µM) was added. All compounds were applied by bath perfusion. Individual neurons were traced and [Ca^2+^]_i_ kinetics (F340/F380 nm) recorded (for some experiments data was collected at 0.2 Hz for a minimum of 3 min; Fig. 1a, c).

For pharmacological experiments, cells were pre-treated with either 100 ng/ml Pertussis Toxin (PTX), 5 µM U-73122, or 1 µM Xestospongin C (XeC), for 5 min in Krebs–Ringer’s solution (for PTX 10 min) before applying LPA. For experiments with VGCC inhibitors LPA was applied, followed by a pre-treatment with either 0.2 µM ω-Agatoxin TK, 10 µM Nifedipine, 0.1 µM SNX-482 or 500 nM ω -Conotoxin-GVIA for 5 min in Krebs–Ringer’s solution before applying a second stimulus of LPA (together with inhibitors).

Cells were washed with HBSS for at least 1 h before applying a second stimulation (Supplementary Figure S1a). In a subset of experiments, either LPA was added followed by stimulation with 5 µg/ml Thapsigargin (Thg) or stimulation with Thg followed by LPA treatment (Supplementary Figure S1b).

Raw data and images were analysed with LAS X software (except for Fig. 1a, c; Cell^P Software). The background-corrected ratio of fluorescence intensity (F340/380 nm) was calculated. [Ca^2+^]_i_ peaks were calculated by averaging the five highest values of the LPA or glutamate peaks subtracting the baseline from them. Averaged LPA peak values were normalized to glutamate peak values reflecting the maximum calcium increase/response of the cells (except for Fig. 1a, c).

Each experimental group was examined in a minimum of three independent experiments.

### Epifluorescence microscopy and confocal microscopy of cultured neurons

For estimation of exocytotic activity (vesicle fusion) primary hippocampal neurons were cultivated in 12-well-dishes at ~ 80.000 cells per coverslip and transfected at DIV 7–10 with superecliptic pHluorin-synaptophysin (kindly provided by V. Hauke, Freie Universität Berlin, Berlin, Germany), what will be referred to as SynaptopHluorin in the manuscript from now on, by Effectene. Microscopy was performed between DIV 14–16. HBSS with 1 µM tetrodotoxin (TTX) was used for all experiments. Images were taken at 25 °C with a high-resolution CCD camera (F-View II) mounted on an inverse microscope (Olympus IX81) with a 100 × 1.3 NA oil-immersion objective equipped with a GFP filter set (DCLP 505, BP 525/50). After acquisition of the tenth image, LPA was added and data was collected at 0.2 Hz for at least 8 min. Images were analyzed using ImageJ. For approximate tagging [Ca^2+^]_i_ in presynaptic terminals neurons transfected with SynaptopHluorin were loaded with Fura-Red/AM (final concentration 10 µM) for 30 min at 37 °C. Images were acquired with a 100 × 1.3 NA oil-immersion objective and corresponding Fura-Red filter (490 nm). After acquisition of the fifth image, LPA was added and data was collected at 0.2 Hz for at least 4 min. Images were analyzed using Cell^P. ROIs were positioned at the centre of exocytotic hotspots, revealing responding synapses and alteration of fluorescence was determined. ROI size was set between 2 and 4 µm for individual boutons to account for fluorescence decay due to fast lateral diffusion (Granseth et al., 2006).

Confocal images were acquired with Leica TCS SL Laser Scanning Confocal Microscope equipped with a 63 × objective (oil-immersion, 1.4 NA) using the 488-nm line of an argon-ion laser. Background correction and adjustment of brightness and contrast were performed using Leica confocal software.

### Electrophysiology

Primary cultured neurons between DIV 10–15 were transferred to extracellular solution, containing in mM: 124 NaCl, 4 KCl, 3 CaCl_2_, 2 MgCl_2_, 25 HEPES, 10 glucose, or 150 NaCl, 3 KCl, 1 MgCl, 2 CaCl_2_, 35 glucose, adjusted to pH 7.4 with NaOH, and placed under an inverse (Axiovert S100) or an upright microscope (AxioscopeFS2mot). Pipette solution comprised (in mM) 120 K gluconate, 10 KCl, 10 Na-phosphocreatine, 1 MgCl_2_, 1 CaCl_2_, 11 EGTA, 10 HEPES, 2 Mg^2+^ ATP and 0.3 GTP and, for recording IPSCs, 145 CsCl, 1 CaCl_2_, 10 EGTA, 20 HEPES, 5 NaCl, 2 MgCl_2_, pH 7.2. Synaptic activity was recorded at –60 or –70 mV at 25 °C using an EPC8 or EPC10 amplifier. Pharmacological isolation of miniature EPSCs (mEPSCs) and mIPSCs was achieved by 0.5 to 1 µM TTX, DL-aminophosphonovaleric acid (100 µM), strychnine (0.4 µM) and their identity confirmed in some experiments by application of excitatory (20 µM DNQX or CNQX) or inhibitory synaptic blockers (2 µM GABAzine or 10 µM bicuculline) (Figure S1e). Signals were filtered at 3 kHz and sampled at a rate of 6.25 to 10 kHz using WinTida or Patchmaster. Postsynaptic currents were analyzed using MiniAnalysis (synaptosoft) or in-house software written by C. Henneberger. To suppress [Ca^2+^]_i_ elevations, CaCl_2_ was omitted and EGTA replaced by BAPTA in the indicated experiments.

### Model description

For modelling LPA effects on the level of the synapse we adapted the single-pool, four-state model developed by [48] which describes the dynamic within a synapse observed with dye-loaded vesicles. We excluded the parameter describing the dye loss dynamics, which resulted in a new single-pool three-state model with: u1: Fraction of vesicles ready for release; u2: Fraction of vesicles currently activated/merged with the pre-synaptic membrane; u3: Fraction of vesicles currently being recycled after endocytosis (empty vesicles not yet in the pool of vesicles that can be released). The dynamics of the model are described with three parameters: α—rate of activation/exocytosis; β—recycling rate (after endocytosis back to the pool of vesicles ready for release); σ—vesicle endocytosis after release. These were set to the values obtained by [48]: α = 0.008 s-1, β = 0.5 s-1, σ = 1.67 s-1, and the dynamics are described with:

$$\frac{{du_{1} }}{dt}\, = \, - \alpha \,*\,u_{1} { + }\beta {\text{*u}}_{{3}}$$

$$\frac{{du_{2} }}{dt}\, = + \alpha *\,u_{1} - \sigma *\,u_{2}$$

$$\frac{{du_{3} }}{dt}\, = \, + \sigma *u_{2} \, - \beta *u_{3}$$

To simulate the effect of LPA we increased the exocytosis rate α by a factor of 1.5 and reduced the rate of recycling β by 0.01. The reduction of the recycling rate was set to start 60 s before the increase of exocytosis. We implemented this model in the scientific programming language Julia ([1209.5145] Julia: A Fast Dynamic Language for Technical Computing (arxiv.org)). To simulate the effect of LPA we increased the exocytosis rate α by a factor of 1.5 and reduced the rate of recycling β by 0.01 to match the observed rate of mEPSC reduction. The reduction of the recycling rate was set to start 60 s before the increase of exocytosis. The full source code script to run the model and generate the data/figures is available at the authors github repository: https://github.com/konstantinstadler/brandt_lpa_neuronal_modulation.

### Data presentation and statistical analysis

All n numbers of the corresponding experiments are listed in Supplementary Table S1. Values were analyzed for normal distribution using the D’Agostino-Pearson omnibus and Shapiro–Wilk test. Statistical significance was determined by means of Students’ *t*-test independent or paired as appropriate when the data were normally distributed. Statistical analysis was performed using non-parametric tests as the data were not normally distributed. When comparing two groups we used Wilcoxon matched-pairs signed rank test for paired or Mann–Whitney U test for unpaired groups. The comparison for more than two groups was performed with the Kruskal–Wallis test with Dunn’s multiple comparison post hoc test. P values < 0.05 were considered to indicate statistical significance. Data analysis was performed using GraphPad Prism7, Origin 7 and Microsoft Excel and visualized with CorelDRAW 2017. Data are presented as mean ± SEM if not noted otherwise and listed in Supplementary Table S1 with p values.

## Supplementary Information

Additional file 1. Figure S1. (a) LPA induces increased intracellular Ca^2+^ levels. Fura-2/AM imaging reveal that application of 10 µM LPA stimulated [Ca^2+^]_i_ increase. The LPA induced [Ca^2+^]_i_ increase develops within seconds and was transient. [Ca^2+^]_i_ gradually returned to its pre-stimulation level and a second stimulation resulted in the same signal intensity. A representative trace of [Ca^2+^]_i_ responses of one neuron is shown here. Arrows indicate the time of LPA pipetting. (b) LPA induces release of Ca^2+^ ions from the endoplasmic reticulum. Fura-2/AM measurements in primary hippocampal neurons stimulated with LPA (10 µM), Thg (5 µg/ml) or L-Glu (100 mM). Representative calcium imaging traces showing the effect of averaged single neuron LPA and Thg applications* (left)* and Thg, LPA and Glu (*right*), respectively. Arrows indicate the time of application. *Left:* Application of LPA triggers [Ca^2+^]_i_ increase, a subsequent stimulus with Thg reveals a similar [Ca^2+^]_i_ increase. *Right:* After depleting ER calcium stores with Thg, raising the [Ca^2+^]_i_, application of LPA could not increase [Ca^2+^]_i_, whereas glutamate still stimulated the[Ca^2+^]_i_ increase. For n numbers see Table S1.(c) *Left: *Time course of experiments in which LPA receptors were inihibited with the specific inhibitor Ki16425 revealed a complete block of an LPA mediated effect on synaptic transmission (*open squares*). In contrast, 1 µM LPA was able to induce a reduction in post-synaptic currents (*filled squares*). *Right*: Summary plot of the experiment after application of 1 µM LPA and during simultaneous application of LPA and Ki16425. For n numbers, statistics and p values see Table S1. (d) Bar graph showing the input resistance of neurons before and after the application of 10 µM LPA. (e) *Left*: Representative recordings from isolated mEPSCs in the presence of TTX and GABA_A_ inhibitors. Subsequent application of glutamatergic blockers to inhibit remaining AMPA receptors abolished all synaptic input to the neuron. Recordings were performed in the presence of 2 mM Mg^2+^ to reduce NMDA receptor activation at the recording potential. *Right*: Example recording of pharmacologically isolated mIPSCs at a holding potential of – 70 mV in the presence of TTX and CNQX. Subsequent wash in of a GABA_A_ receptor blocker abolished all inhibitory synaptic currents. For n numbers see Table S1

Additional file 2.

Additional file 3.

## Data Availability

The raw data supporting the conclusions of this article will be made available by the authors, without undue reservation.
