# Supplementary figures and images for: Lysophosphatidic acid selectively modulates excitatory transmission in hippocampal neurons
Source: Cell Biosci. 2025 Aug 12;15:117. doi: 10.1186/s13578-025-01458-y (PMC12341218; doi:10.1186/s13578-025-01458-y)

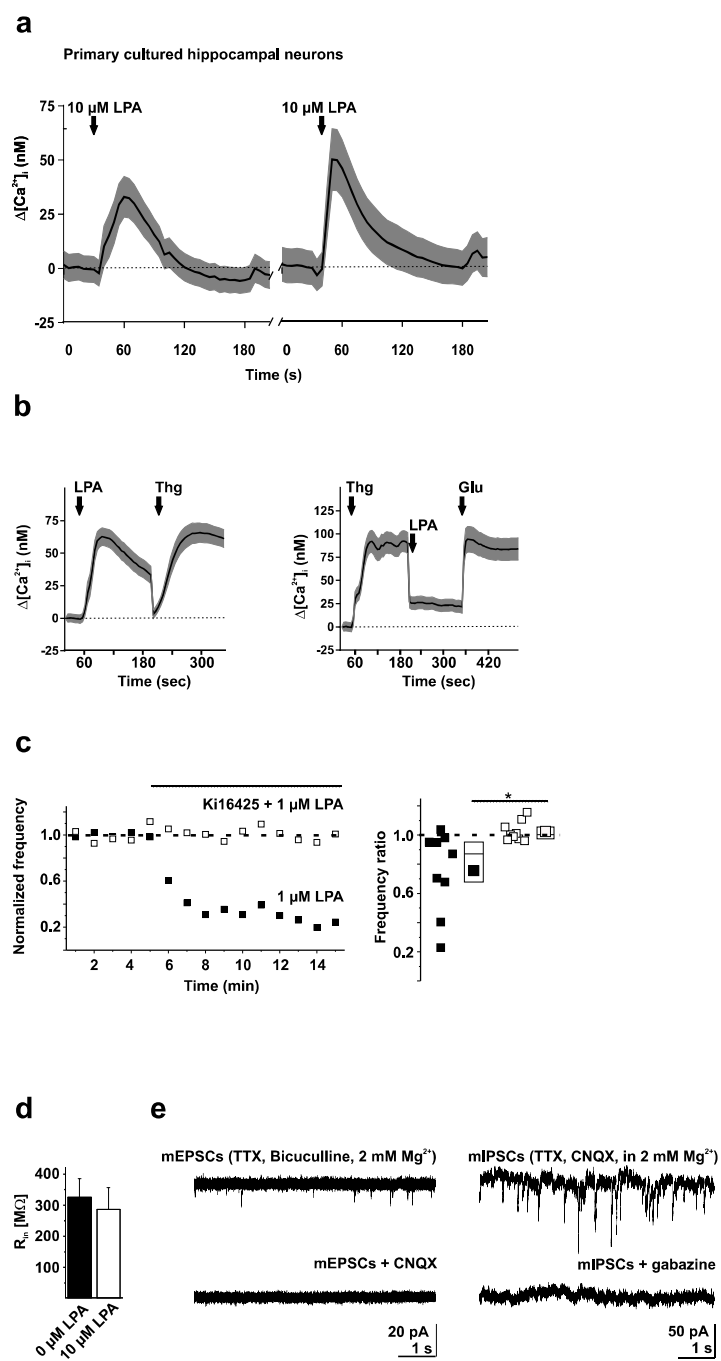

Supplement: Supplementary file 1 — Additional file 1. Figure S1. (a) LPA induces increased intracellular Ca2+ levels. Fura-2/AM imaging reveal that application of 10 µM LPA stimulated [Ca2+]i increase. The LPA induced [Ca2+]i increase develops within seconds and was transient. [Ca2+]i gradually returned to its pre-stimulation level and a second stimulation resulted in the same signal intensity. A representative trace of [Ca2+]i responses of one neuron is shown here. Arrows indicate the time of LPA pipetting. (b) LPA induces release of Ca2+ ions from the endoplasmic reticulum. Fura-2/AM measurements in primary hippocampal neurons stimulated with LPA (10 µM), Thg (5 µg/ml) or L-Glu (100 mM). Representative calcium imaging traces showing the effect of averaged single neuron LPA and Thg applications (left) and Thg, LPA and Glu (right), respectively. Arrows indicate the time of application. Left: Application of LPA triggers [Ca2+]i increase, a subsequent stimulus with Thg reveals a similar [Ca2+]i increase. Right: After depleting ER calcium stores with Thg, raising the [Ca2+]i, application of LPA could not increase [Ca2+]i, whereas glutamate still stimulated the[Ca2+]i increase. For n numbers see Table S1.(c) Left: Time course of experiments in which LPA receptors were inihibited with the specific inhibitor Ki16425 revealed a complete block of an LPA mediated effect on synaptic transmission (open squares). In contrast, 1 µM LPA was able to induce a reduction in post-synaptic currents (filled squares). Right: Summary plot of the experiment after application of 1 µM LPA and during simultaneous application of LPA and Ki16425. For n numbers, statistics and p values see Table S1. (d) Bar graph showing the input resistance of neurons before and after the application of 10 µM LPA. (e) Left: Representative recordings from isolated mEPSCs in the presence of TTX and GABAA inhibitors. Subsequent application of glutamatergic blockers to inhibit remaining AMPA receptors abolished all synaptic input to the neuron. Reco [file 13578_2025_1458_MOESM1_ESM.pdf]
