# Supplementary material for: Lysophosphatidic acid selectively modulates excitatory transmission in hippocampal neurons
Source: Cell Biosci. 2025 Aug 12;15:117. doi: 10.1186/s13578-025-01458-y (PMC12341218; doi:10.1186/s13578-025-01458-y)
Supplement: Supplementary file 2 — Additional file 2. [file 13578_2025_1458_MOESM2_ESM.docx]

**Supplementary Table 1: means±SEM/SD and summary of p values of data presented**

| **Figure** | **Condition** | **means± SD** | **means± SEM** | **n** | **Comparison** | **Statistics** | **p value** |
| --- | --- | --- | --- | --- | --- | --- | --- |
| **1a** | **Δ[Ca^2+^]_i_ (nM)** |  |  |  |  |  |  |
|  | 0.1 | 0±0 |  | 26 |  |  |  |
|  | 0.5 | 35.4±25.7 |  | 18 |  |  |  |
|  | 1 | 52.94±39.6 |  | 26 |  |  |  |
|  | 5 | 262.7±109.7 |  | 42 |  |  |  |
|  | 10 | 147.8±79.6 |  | 47 |  |  |  |
|  | 50 | 125.4±55.9 |  | 55 |  |  |  |
| **1c** | **Δ[Ca^2+^]_i_ (nM)** |  |  |  |  |  |  |
|  | LPA_2_R^-/-^ |  |  | 57 |  |  |  |
| **1e/f** | **Δ[Ca^2+^]_i_** |  |  |  |  |  |  |
|  | LPA |  | 62.4±3.1 | 135 |  |  |  |
|  | PTX |  | 1 ±0.2 | 46 | LPA vs. PTX | Mann-Whitney U test; unpaired | p<0.0001 |
|  | U73122 |  | 10.8±2.4 | 40 | LPA vs. U73122 | Mann-Whitney U test; unpaired | p<0.0001 |
|  | XeC |  | 5.3±1.3 | 66 | LPA vs. XeC | Mann-Whitney U test; unpaired | p<0.0001 |
| **1g** | **Δ[Ca^2+^]_i_** |  |  |  |  |  |  |
|  | LPA |  | 45.1±2 | 100 | LPA vs. LPA+ ω -Agatoxin TK | Wilcoxon-signed-rank test; paired | p<0.0001 |
|  | LPA+ ω -Agatoxin TK |  | 6.3±1.2 | 100 |  |  |  |
|  | LPA |  | 53±1.5 | 166 | LPA vs. LPA+ ω -Conotoxin-GVIA | Wilcoxon-signed-rank test; paired | p<0.0001 |
|  | LPA+ ω -Conotoxin-GVIA |  | 6.6±1 | 166 |  |  |  |
|  | LPA |  | 27.6±1 | 174 | LPA vs. LPA+ Nifedipine | Wilcoxon-signed-rank test; paired | p<0.0001 |
|  | LPA+Nifedipine |  | 21.3 ± 1 | 174 |  |  |  |
|  | LPA |  | 45.5 ± 1.2 | 172 | LPA vs. LPA+ Nifedipine | Wilcoxon-signed-rank test; paired | p<0.0001 |
|  | LPA+SNX-482 |  | 37.1±1.7 | 172 |  |  |  |
| **1h** | **Δ[Ca^2+^]_i_ in % normalized to LPA** |  |  |  |  |  |  |
|  | LPA+ ω -Agatoxin TK |  | 21.8 ± 5.8 | 100 |  | Kruskal-wallis test; unpaired | p<0.0001 |
|  | LPA+ ω -Conotoxin-GVIA |  | 11.9±1.6 | 166 |  | Kruskal-wallis test; unpaired | p<0.0001 |
|  | LPA+Nifedipine |  | 84.7 ± 4.4 | 174 |  | Kruskal-wallis test; unpaired | p<0.0001 |
|  | LPA+SNX-482 |  | 76.1 ± 3.2 | 172 |  | Kruskal-wallis test; unpaired | p<0.0001 |
|  |  |  |  |  | LPA+ ω -Agatoxin TK vs. LPA+ ω -Conotoxin-GVIA | Kruskal-wallis test; Dunn’s multiple comparisons test | p>0.9999 |
|  |  |  |  |  | LPA+ ω -Agatoxin TK vs. LPA+Nifedipine | Kruskal-wallis test; Dunn’s multiple comparisons test | p<0.0001 |
|  |  |  |  |  | LPA+ ω -Agatoxin TK vs. LPA+SNX-482 | Kruskal-wallis test; Dunn’s multiple comparisons test | p<0.0001 |
|  |  |  |  |  | LPA+ ω -Conotoxin-GVIA vs. LPA+Nifedipine | Kruskal-wallis test; Dunn’s multiple comparisons test | p<0.0001 |
|  |  |  |  |  | LPA+ ω -Conotoxin-GVIA vs. LPA+SNX-482 | Kruskal-wallis test; Dunn’s multiple comparisons test | p<0.0001 |
|  |  |  |  |  | LPA+Nifedipine vs. LPA+SNX-482 | Kruskal-wallis test; Dunn’s multiple comparisons test | p>0.9999 |
| **2a** | Diagram shows mean ± SEM of n= 4 ROI |  |  | 78 |  |  |  |
| **2b** | **mEPSC frequency** |  |  |  |  |  |  |
|  | LPA:ctrl |  | 3.4 ± 0.95 s^-1^ : 2.4 ± 0.75 s^-1^ = 0.61 | 13 | w/o LPA vs. w LPA | Wilcoxon signed rank test | p = 0.0002 |
|  | **mIPSC frequency** |  |  |  |  |  |  |
|  | LPA:ctrl |  | 1.6 ± 0.7 s^-1^ : 1.6 ± 0.7 s^-1^ = 1.01:1 | 10 | w/o LPA vs. w LPA | Wilcoxon signed rank test | p = 0.85 |
| **2c** | **mEPSC amplitude** |  |  |  |  |  |  |
|  | w/o LPA |  | 28.3 ± 2.9 pA | 13 | w/o LPA vs. w LPA | paired t-test | p = 0.2 |
|  | w LPA |  | 26.6 ± 3.1 pA | 13 |  |  |  |
|  | **mIPSC amplitude** |  |  |  |  |  |  |
|  | w/o LPA |  | 31.3 ± 1.5 pA | 10 | w/o LPA vs. w LPA | paired t-test | p = 0.24 |
|  | w LPA |  | 29.7 ± 2.0 pA | 10 |  |  |  |
| **2d** | **Number of vesicles** |  |  |  |  |  |  |
|  | **Asymmetric terminals** |  |  |  |  |  |  |
|  | w/o LPA |  | 33 ± 2.7 | 21 | w/o LPA vs. w LPA | t-test; unpaired | p = 0.0048 |
|  | w LPA |  | 22.4 ± 2.3 | 22 |  |  |  |
|  | **Symmetric terminals** |  |  |  |  |  |  |
|  | w/o LPA |  | 34 ± 2.4 | 26 | w/o LPA vs. w LPA | Mann-Whitney U test; unpaired | p = 0.35 |
|  | w LPA |  | 30.9 ± 2.6 | 26 |  |  |  |
| **3a** | **LPA_2_R** |  |  |  |  |  |  |
|  | Symmetric synapses |  | 1.01 ± 0.06 | 15 | Symmetric vs. Asymmetric synapses | t-test; unpaired | p =0.006 |
|  | Asymmetric synapses |  | 1.25±0.05 | 18 |  |  |  |
| **3b** | **mEPSC frequency** |  |  |  |  |  |  |
|  | LPA:ctrl (LPA_2_R^-/-^) |  | 5.3 ± 1.8 s^-1^ : 5.4 ± 1.9 s^-1^ = 0.98:1 | 6 | LPA vs. ctrl (LPA_2_R^-/-^) | paired t-test | p = 0.45 |
| **3c** | **mEPSC frequency** |  |  |  |  |  |  |
|  | **0 Ca^2+^extracellular** |  |  |  |  |  |  |
|  | LPA:ctrl |  | 2.8 ± 1 s^-1^ : 2.9 ± 1 s^-1^ = 0.93 | 7 |  | Wilcoxon signed rank test | p = 0.24 |
| **3d** | **BAPTA intracellular** |  |  |  |  |  |  |
|  | LPA:ctrl |  | 2.9 ± 1.1 s^-1^ : 4 ± 1.3 s^-1^ = 0.72 | 9 |  | paired t-test | p *=* 0.002 |
| **4b** | **cumulative vesicle** |  |  |  |  |  |  |
|  | LPA WT |  |  | 10 |  |  |  |
|  | LPA_2_R^-/-^ |  |  | 4 |  |  |  |
| **4c** | **Fluorescence decay time** |  |  |  |  |  |  |
|  | LPA τ |  | 122 ±12.2 | 36 | LPA τ vs. L-Glu τ | Mann-Whitney U test; unpaired | p < 0.0001 |
|  | L-Glu τ |  | 43.7 ± 4.4 | 35 |  |  |  |
| **4d** | **mEPSC frequency** |  |  |  |  |  |  |
|  | LPA:ctrl |  | 8.1 ± 2.6 s^-1^ : 7.1 ± 2.4 s^-1^ = 1.21 | 10 | LPA vs. ctrl | Wilcoxon signed rank test | p < 0.005 |
|  | **mEPSC amplitude** |  |  |  |  |  |  |
|  | w/o LPA |  | 20.7 ± 2.9 pA | 10 |  |  |  |
|  | LPA |  | 20.4 ± 2.9 pA | 10 |  | paired t-test | p = 0.3 |
| **Suppl. Figure 1b** | Δ **[Ca2+]i in nM** |  |  |  |  |  |  |
|  | Thapsigargin |  |  | 17 (left panel) / 28 (right panel) |  |  |  |
|  | LPA |  |  | 17 (left panel) / 28 (right panel) |  |  |  |
|  | L-glutamate |  |  | 28 (right) |  |  |  |
| **Suppl. Figure 1c** | **mEPSC frequency** |  |  |  |  |  |  |
|  | LPA:ctrl |  | 0.75:1 | 9 | LPA vs. ctrl | paired t-test | p < 0.05 |
|  | LPA:ctrl (KI16425) |  | 1.02:1 | 9 | LPA vs. ctrl | paired t-test | p = 0.35 |
| **Suppl. Figure 1d** | **Input resistance (R_in_)** |  | Ctrl: 325.4 ± 60 MΩ  LPA: 286.9 ± 70 MΩ | 10 | LPA vs. ctrl | Wilcoxon signed rank test | p = 0.32 |
