# Supplementary material for: Lysophosphatidic acid selectively modulates excitatory transmission in hippocampal neurons
Source: Cell Biosci. 2025 Aug 12;15:117. doi: 10.1186/s13578-025-01458-y (PMC12341218; doi:10.1186/s13578-025-01458-y)
Supplement: Supplementary file 3 — Additional file 3. [file 13578_2025_1458_MOESM3_ESM.docx]

**Supplementary Table 2: Key Resources**

| **Reagent or Resource** | **Source** | **Identifier** |
| --- | --- | --- |
| Antibodies | | |
| anti-LPA_2_ (monoclonal rat) | Dr. J. Aoki |  |
| anti-rat biotin-conjugated | Molecular Probes, Eugene, OR, USA |  |
| Goat anti-rat 488 | Molecular Probes, Eugene, OR, USA |  |
| Bacterial and Virus Strains | | |
| superecliptic pHluorin-synaptophysin (pFUGW-SytI) | kindly provided by V. Hauke, Freie Universität Berlin, Berlin, Germany |  |
| **Biological Samples** |  |  |
| none |  |  |
| Chemicals, Peptides, and Recombinant Proteins | | |
| Monounsaturated 1-Oleoyl-lysophosphatidic acid (18:1, or LPA) | Sigma-Aldrich Chemie, Steinheim, Germany | Cat#7260 |
| HBSS (Hank's Buffered Salt Solution) | Thermo Fisher Scientific, Dreieich, Germany | Cat#14170 |
| MEM (Modified Eagles Medium) | Thermo Fisher Scientific, Dreieich, Germany | Cat#31095029 |
| NBA (Neurobasal ^TM^ A medium) | Thermo Fisher Scientific, Dreieich, Germany | Cat#21103049 |
| horse serum | Thermo Fisher Scientific, Dreieich, Germany | Cat#26050088 |
| FCS (Fetal calf serum) | Pan Biotech, Aidenbach, Germany | Cat#P30-3306 |
| Penicillin-Streptomycin | Pan Biotech | Cat#P06-07050 |
| B27^TM^ Supplement | Thermo Fisher Scientific, Dreieich, Germany | Cat#17504044 |
| poly-L-lysine | Sigma-Aldrich Chemie, Steinheim, Germany | Cat#P2636 |
| L-glutamine | Sigma-Aldrich Chemie, Steinheim, Germany | Cat#59202C |
| Trypsin | Thermo Fisher Scientific, Dreieich, Germany | Cat#11538876 |
| Fura-2 AM | Thermo Fisher Scientific, Dreieich, Germany | Cat#F1221 |
| Fura Red AM | Thermo Fisher Scientific, Dreieich, Germany | Cat#F3021 |
| Pertussis Toxin (PTX) | Calbiochem, San Diego, CA, USA | Cat#516560 |
| U-73122 | Calbiochem, San Diego, CA, USA | Cat#662035 |
| Xestospongin C (XeC) | Biomol, Plymouth Meeting, PA, USA | Cat#Cay-64950 |
| omega-Agatoxin TK | Abcam, Cambridge, UK | Cat#ab141780 |
| Nifedipine | Tocris; Bristol, UK | Cat#1075/100 |
| SNX-482 | Alomone labs, Jerusalem, Israel | Cat#RTS-500 |
| omega-Conotoxin-GVIA | AnaSpec, San Jose, CA, USA | AS-22926 |
| TTX citrate | Tocris; Bristol, UK | CAS 18660-81-6 |
| DL‑aminophosphonovaleric acid | Sigma-Aldrich Chemie, Steinheim, Germany | CAS 79055-68-8 |
| Strychnine hydrochloride | Sigma-Aldrich Chemie, Steinheim, Germany | CAS 1421-86-9 |
| CNQX | Tocris; Bristol, UK | CAS 479347-85-8 |
| Gabazine (SR95531 hydrobromide) | Tocris; Bristol, UK | CAS 104104-50-9 |
| Bicuculline methiodide | Tocris; Bristol, UK | CAS 40709-69-1 |
| 3, 3’-diaminobenzidine | Vector Laboratories, Burlingame, CA, USA | SK4100 |
| Critical Commercial Assays | | |
| Effectene transfection reagent | Qiagen, Hilden, Germany | Cat#301427 |
| Deposited Data | | |
| none |  |  |
| Experimental Models: Cell Lines | | |
| none |  |  |
| Experimental Models: Organisms/Strains | | |
| Mouse: C57/Bl6 wild-type mice | central animal facility |  |
| Mouse: BalbC wild-type mice | central animal facility |  |
| Mouse: LPA_2_R^–/–^ | Dr. J. Chun | Contos *et al.*, 2002 |
| Oligonucleotides | | |
| none |  |  |
| Recombinant DNA | | |
| none |  |  |
| Software and Algorithms | | |
| LAS X software | Leica, Wetzlar, Germany |  |
| GraphPad Prism7 | GraphPad Software Inc., La Jolla, CA, USA |  |
| Microsoft Excel |  |  |
| Cell^P Software | Olympus |  |
| MiniAnalysis 6.0.9. | Synaptosoft, Fort Lee, NJ, USA |  |
| Image J | Schneider *et al.*, 2012 |  |
| SPSS Statistics 17.0 Software | SPSS Statistics for Windows, Version 17.0. Chicago: SPSS Inc., IL, USA |  |
| Origin 7 | OriginLab Corporation, NH, USA |  |
| other | | |
| Olympus IX70 microscope equipped with a calcium imaging system | Olympus,  Till-photonics, Munich, Germany |  |
| perfusion chamber (RC-21B) | Warner Instrument Corporation, Hamden, CT, USA |  |
| Leica DMi8 microscope equipped with a calcium imaging system: HC PL FLUOTAR 40x/1.30 Oil (Fura) or HC PL FLUOTAR 20x/0.80 Oil (Fura) objectives  Fura2 Filter set (Excitation: 340/26, 380/11, DC: 400, Emission: 510, 80) | Leica, Wetzlar, Germany |  |
| X-Cite 200 DC illuminator (Calcium imaging) | Excelitas Technologies, Mississauga ON, Canada |  |
| PPS2 Peristaltic Perfusion System (Calcium Imaging) | Multi Channel Systems, Reutlingen, Germany |  |
| Leica DFC9000 GT Camera (Calcium imaging) | Leica, Wetzlar, Germany |  |
| CCD camera F-View II (Calcium Imaging SynaptopHluorin) | Soft Imaging System GmbH, Münster, Germany |  |
| inverse microscope IX81 | Olympus |  |
| Axiovert S100 | Zeiss, Oberkochen, Germany |  |
| AxioscopeFS2mot | Zeiss, Oberkochen, Germany |  |
| EPC8 or EPC10 amplifier | HEKA, Lambrecht, Germany |  |
| WinTida4.11 | HEKA, Lambrecht, Germany |  |
| Patchmaster2.3 | HEKA, Lambrecht, Germany |  |
| MiniAnalysis 6.0.9 | Synaptosoft, Fort Lee, NJ, USA |  |
